# A motion-energy model predicts the direction discrimination and MAE duration of two-stroke apparent motion at high and low retinal illuminance

**DOI:** 10.1016/j.visres.2010.04.002

**Published:** 2010-06-11

**Authors:** Kirsten L. Challinor, George Mather

**Affiliations:** School of Psychology, University of Sussex, Falmer, Brighton BN1 9QH, UK

**Keywords:** Two-stroke apparent motion, Motion energy, Scotopic, Temporal impulse response, Motion after-effect

## Abstract

Two-stroke apparent motion offers a challenge to current theoretical models of motion processing and is thus a useful tool for investigating motion sensor input. The stimulus involves repeated presentation of two pattern frames containing a spatial displacement, with a blank inter-stimulus interval (ISI) at one of the two-frame transitions. The resulting impression of continuous motion was measured here using both direction discrimination and motion after-effect duration in order to assess the extent to which data using the two measures can be explained by a computational model without reference to attentive tracking mechanisms. The motion-energy model was found to offer a very good account of the psychophysical data using similar parameters for both tasks. The experiment was run under both photopic and scotopic retinal illumination. Data revealed that the optimum ISI for perceiving two-stroke apparent motion shifts to longer ISIs under scotopic conditions, providing evidence for a biphasic impulse response at low luminance. Best-fitting model parameters indicate that motion sensors receive inputs from temporal filters whose central temporal frequency shifts from 2.5 to 3.0 Hz at high retinal illuminance to 1.0–1.5 Hz at low retinal illuminance.

## 1 Introduction

**Fig. 1:**
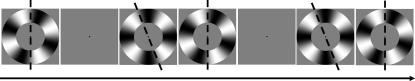
Schematic of the two-stroke apparent motion sequence. Two pattern frames with a 90° phase difference are presented repeatedly. An inter-stimulus interval (ISI) occurs at one of the frame transitions. This example appears to move continuously in a clockwise direction. To aid clarity, the figure includes a superimposed dashed line indicating spatial phase, and is shown at higher contrast and an eighth of the spatial frequency used by the experimental stimulus in the current study.

Certain kinds of dynamic visual stimuli provoke consistent errors in reports of perceived motion direction. Such stimuli are especially interesting from a theoretical point of view because they shed light on the properties of the underlying neural processes serving motion perception, and offer a challenge to theoretical models. In this paper we present two psychophysical measures of one such motion stimulus, known as two-stroke apparent motion, and examine the ability of the dominant current model of low-level motion processing to explain the data. An example of the two-stroke apparent motion stimulus sequence is illustrated in Fig. 1. An annular grating rotates by one quarter-cycle from frame 1 to frame 2, and is interrupted by a brief, uniform inter-stimulus interval (ISI). When the sequence is repeated observers report an impression of continuous clockwise rotation, even though the grating oscillates between only two positions (Mather, 2006). In a previous paper (Mather & Challinor, 2009) we reported that adaptation to the sequence in Fig. 1 generates a motion after-effect (MAE).

In this paper we provide new psychophysical data to compare the MAE measure against another common measure of motion perception, namely reported direction in a direction discrimination task. MAEs are widely accepted as providing a direct perceptual measure of changes in neural activity (Mather, Pavan, Campana, & Casco, 2009; Mather, Verstraten, & Anstis, 1998). On the other hand, a number of papers have reported that direction discrimination data may not offer a pure measure of motion sensor output (e.g. Bex & Baker, 1999; Boulton & Baker, 1993; Takeuchi & De Valois, 2009; Ukkonen & Derrington, 2000). In particular, Takeuchi and De Valois (2009) studied errors in a direction discrimination task using two pattern frames separated by an ISI. In a previous paper they had reported that errors are maximal at ISIs of around 30 ms (Takeuchi & De Valois, 1997). But more recently (Takeuchi & De Valois, 2009) they report that in centrally viewed stimuli attentive tracking can be deployed, removing direction discrimination errors. Motion reversals of the kind reported in Takeuchi and De Valois’s papers are thought to be a component of two-stroke apparent motion, because in an earlier paper (Mather & Challinor, 2009) we found that MAEs from two-stroke apparent motion are maximal for ISIs lasting approximately 30 ms.

Although our stimuli are annular gratings which avoid central viewing, other research indicates that such stimuli can support attentive tracking (Cavanagh, 1992), so it remains a possibility that attentive tracking influences direction discrimination performance using two-stroke stimuli. We therefore decided to compare data on MAE duration against direction discrimination data using the same annular two-stroke stimulus, at both high and low retinal illuminance. Measurements were taken at a range of ISIs from 0 to 317 ms, for both photopic and scotopic vision.

A second aim of the research was to assess the extent to which psychophysical data gathered using the two measures can be explained quantitatively by a computational model of motion sensor output. The Adelson and Bergen (1985) spatiotemporal energy model has become a standard theoretical framework for low-level motion analysis. Georgeson and Scott-Samuel (1999) updated the model based on both physiological and psychophysical evidence (Emerson, Bergen, & Adelson, 1992; Georgeson & Scott-Samuel, 1999; Heeger, 1993) to include opponent motion normalisation. The simple modification involves dividing the Adelson and Bergen output, Opponent Energy, by Flicker Energy, where Opponent Energy is defined as the difference between motion energy moving to the left and moving to the right, and Flicker Energy is the sum of total motion energy. The resulting metric is called ‘Motion Contrast’ and predicts psychophysical performance on direction discrimination tasks (Georgeson & Scott-Samuel 1999).

**Fig. 2:**
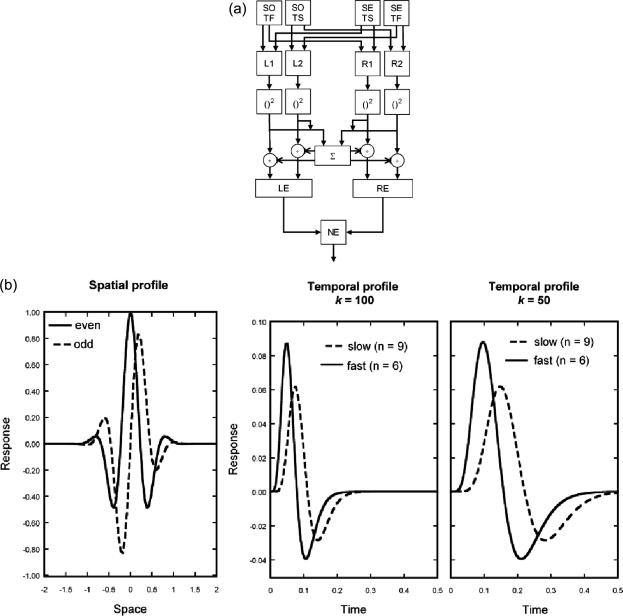
(a) The elaborated Adelson and Bergen (1985) energy model see text for details. (b) Filter profiles used in the model. Left: Odd and even model spatial filters. Middle: Slow and fast temporal filters for when model parameter *k* is 100, and also for when *k* is set to 50 (right).

We implemented an extended version of the spatiotemporal energy model equivalent to that proposed by Georgeson and Scott-Samuel (1999). The motion contrast computation proposed by Georgeson and Scott-Samuel (1999) is computationally equivalent to the response normalisation described in the physiological literature (Emerson et al., 1992; Heeger, 1993). Fig. 2a illustrates the sequence of operations in the extended model. Four energy sensors (two for rightwards motion, and two for leftwards motion) are constructed by summing appropriate combinations of two spatial filters (odd and even, SO and SE, as in Fig. 2b left) and two temporal filters (fast and slow, both biphasic, TF and TS, as in Fig. 2b middle and right). Sensor output is squared and then normalised before net energy is computed from the difference between left and right sensor outputs. Normalising before calculating net energy (as in Fig. 2a) is mathematically equivalent to normalising after calculating net energy (as in Georgeson & Scott-Samuel, 1999), here normalisation is calculated first as this best reflects the physiological responses of cells in the visual cortex (Schwartz & Simoncelli, 2001). The original Adelson and Bergen (1985) model offers a good qualitative account of errors in direction discrimination in two-frame displays separated by an ISI (Takeuchi & De Valois, 2009), but it remains to be established whether a more plausible form of the energy model can offer an adequate explanation of MAE and direction discrimination data obtained using two-stroke motion displays.

## 2 Methods

### 2.1 Psychophysics

#### 2.1.1 Participants

Five observers participated in the experiment measuring direction discrimination performance, one author and four postgraduates who were naïve to the purpose of the experiment. Three of these observers also took part in the experiment which measured MAE duration, along with two other naïve observers. All wore appropriate optical correction as required. The study conformed to the requirements of the University of Sussex Research Governance Committee.

#### 2.1.2 Apparatus

Stimuli were displayed on a Mitsubishi Diamond Pro 2070 monitor at a frame rate of 120 Hz using a ViSaGe stimulus generator controlled by a Dell PC running Matlab 7.5.0 (R2007b). The mean luminance of the 1024 × 768 pixel monitor was 45.99 cd/m^2^. The display was gamma corrected to ensure linearity. Responses were collected using a Cedrus RB530 response box.

#### 2.1.3 Stimuli

Stimuli were radial sine-wave gratings presented within an annulus against a uniform grey background (45.99 cd/m^2^). The inner and outer diameters of the annulus were 4.32° and 8.64° respectively, resulting in approximately 6.5° of visual angle between the small, dark, central fixation point and the mid-point of the annulus. The grating had an equivalent linear spatial frequency of 1.6 cpd (measured around the mid-point of the annulus) and a contrast of 0.5.

For the direction discrimination measurements a seven-frame animation sequence was shown in each trial, as follows: Grating 1 – ISI – Grating 2 – Grating 1 – ISI – Grating 2 – Grating 1. Grating 2 was identical to Grating 1 except for a shift in spatial phase of either +90° or −90°. Pattern frame (grating) duration was fixed at 42 ms (5 ViSaGe frames). ISI duration was varied between trials with the following values: 0, 42, 83, 125, 167, 200, 242, 283 & 317 ms, such that total stimulus duration ranged between 208 and 841 ms. For MAE duration measurements, the seven-frame sequence was extended to cycle continuously for a period of 30 s, after which a static test grating was presented for duration measurement. Auditory tones denoted the adaptation and test periods of the experiment.

#### 2.1.4 Procedure

Observers were seated in a dark room 114 cm from the monitor with their chin and head positioned on a rest clamped to the bench. Direction discrimination trials were presented using a one-interval, two alternative forced choice method of constant stimuli. One of nine possible ISI durations was pseudo-randomly selected for presentation in each trial. In half of the seven-frame presentations the grating rotated clockwise (CW), and in the other half the grating rotated counter-clockwise (CCW). Grating direction was selected pseudo-randomly, with the constraint that no more than three consecutive trials employed the same grating direction. The observer’s task was to report the direction of grating motion (CW or CCW) by pressing a response key after each presentation. There were 40 trials presented for each motion direction, which were collapsed together to give a percentage performance measure for each ISI duration for each participant.

For MAE measurements, following 30 s of adaptation the observer indicated the cessation of the after-effect with a key press. There was also a button to indicate when no motion after-effect was seen. A 30 s recovery period followed before the beginning of the next trial. Adapting direction alternated from trial to trial. Trials were presented in pseudo-random order over 2 × 20 min experimental sessions and were interrupted by a 5 min break. Responses of ‘no-motion after-effect’ were assigned a MAE duration of 0 ms. Each observer’s average duration was calculated from four MAE measurements at each of the nine possible ISI durations for each luminance condition.

For measurements in photopic vision, observers viewed the display directly (mean luminance 45.99 cd/m^2^); for measurements in scotopic vision, observers wore large spectacles containing neutral density filters with an attenuation of 2.3 log units, bringing mean display luminance down to 0.23 cd/m^2^. In scotopic conditions subjects were dark-adapted for 20 min before commencing observations.

#### 2.1.5 Computational modelling

**Fig. 3:**
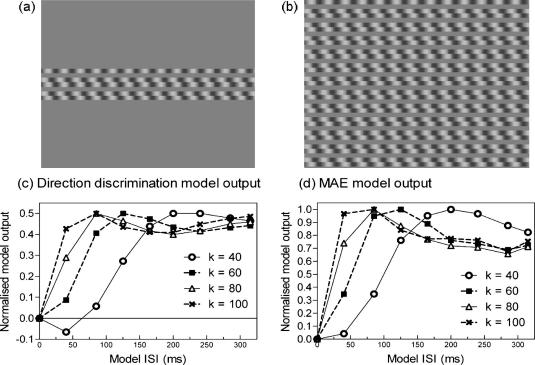
Model stimuli for direction discrimination (a), and MAE (b). The extended motion-energy model output for some exemplar *k* values in response to the direction discrimination stimulus (c) and the MAE stimulus (d).

The model sketched in Fig. 2a was implemented in Matlab. Stimuli and filters were stored as *xt* profiles in Matlab matrices. The spatial dimension of the stimulus luminance profile covered 8° (sampled at intervals of .05°), and the temporal dimension of the stimulus profile extended to 1.5 s (sampled at intervals of .005 s). The direction discrimination model stimulus was a single seven-frame cycle of the two-stroke stimulus within a grey matrix and the MAE stimulus was a repeating cycle of the seven-frame stimulus (Fig. 3a and b). Comparable to the experiment, the model stimulus had a grating frame duration of 40 ms and ISI durations of 0, 40, 85, 125, 165, 200, 240, 285 and 315 ms.

Filter profiles covered 4° of space and 0.5 s of time, at the same sampling rate as the stimulus. Spatial filter profiles were even (*E*) and odd (*O*) Gabor functions:

(1) $$E(x)=\cos(2\pi \mathrm{fx})\cdot {\text{exp}}^{(x/\sigma ){\;}^{2}}$$

(2) $$O(x)=\sin(2\pi \mathrm{fx})\cdot {\text{exp}}^{(x/\sigma ){\;}^{2}}$$

where *f* is 1.1 cpd and *σ* is 0.5°.

Temporal filters had the following form, taken from Adelson and Bergen (1985) Eq. (1):

(3) $$R(t)=(\mathrm{kt}){\;}^{n}\cdot {\text{exp}}^{\left(-\mathrm{kt}\right)}\cdot [1/n!-\beta (\mathrm{kt}){\;}^{2}/(n+2)!]$$

The scale factor, *k*, was varied. It represents the centre temporal frequency of the filter. The effect of manipulating *k* on the model output is shown for some example values in Fig. 3 for both direction discrimination, Fig. 3c, and MAE duration, Fig. 3d. The parameter, *n*, was equal to 9 for the slow temporal filter and six for the fast temporal filter, as used in previous modelling (Emerson et al., 1992; Strout, Pantle, & Mills, 1994; Takeuchi & De Valois, 1997). Fig. 2b shows plots of the spatial and temporal filters used in the modelling. The parameter *β* reflects the weighting of the negative phase of the temporal impulse response relative to the first positive phase and was set to 0.9 (Bergen & Wilson, 1985; Emerson et al., 1992). As shown in Fig. 2a, the output of each of the four energy sensors is divided by the total energy across all sensors. Model output (normalised energy, NE) is then given by subtracting leftward sensor outputs from rightward sensor outputs. Normalisation ensures that NE varies between −1 (all energy leftward) and +1 (all energy rightward).

Although we have not yet implemented adaptation in the computational model it is possible to make predictions for the MAE as follows. Current models of motion adaptation (van de Grind, van der Smagt, & Verstraten, 2004) view it as due to a change in the response gain of motion sensors. Since the change in gain is proportional to the activation level produced by the adapting stimulus (and hence creates an imbalance in response during testing) we can use activity in response to the adapting stimulus as a proxy for the level of adaptation and hence the strength of the resulting MAE.

## 3 Results

### 3.1 Direction discrimination

**Fig. 4:**
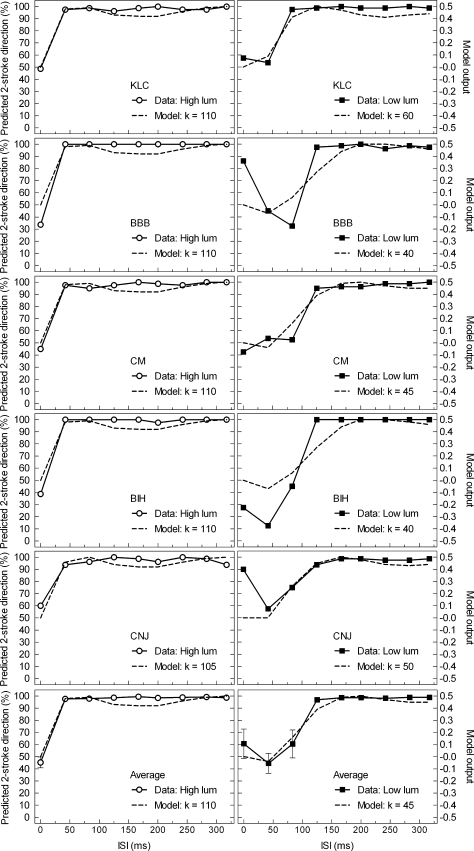
Direction discrimination performance (solid lines) is plotted on the primary *y*-axis as a function of ISI duration for high and low luminance (left column and right column respectively). Performance of 100% occurs when observers perceived continuous apparent motion of the two-stoke stimulus (clockwise motion for the example given in Fig. 1). The first five rows are results for the individual observers and the final row is the mean average of the observers with error bars of ±1 SE of the group mean. The secondary *y*-axis plots the model output (dashed lines) against ISI duration for the best fitting, scale parameter, *k*.

Fig. 4 presents the direction discrimination data for each participant and the group mean (rows) for the light and dark luminance condition (columns). The primary *y*-axis of the plots in Fig. 4 is percentage of reports of continuous motion in the direction predicted by the two-stroke effect. This is the direction for which observers perceive continuous apparent motion of the two-stroke stimulus (clockwise motion for the example given in Fig. 1). This measure of motion is plotted for both photopic (open circles) and scotopic luminances (black squares) as a function of ISI duration in milliseconds. Chance performance is at 50% and was expected to be shown for the 0 ms ISI condition as the stimulus is essentially a grating oscillating through 90° phase shifts left and right. For the photopic condition, an ISI of 42 ms or greater results in the perception of continuous two-stroke motion for all subjects. Under scotopic luminance, the group average gives continuous motion perception for ISIs greater than 125 ms.

**Fig. 5:**
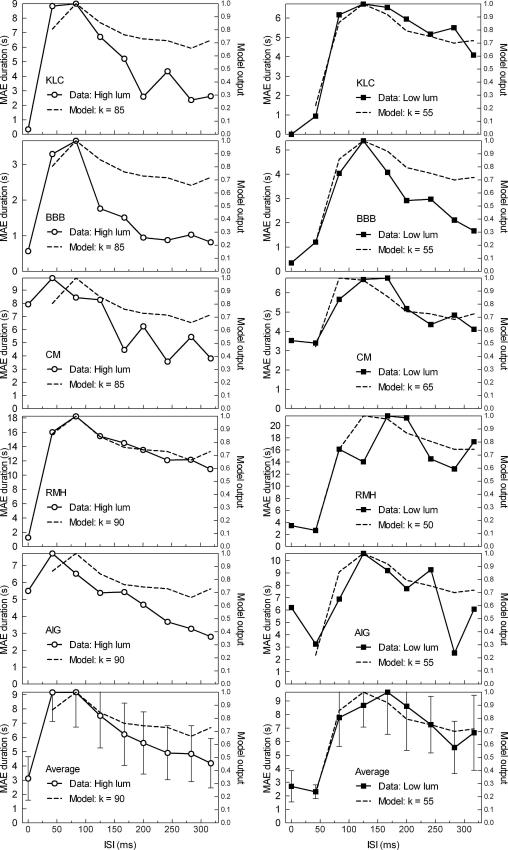
Results for the MAE experiment. Similar to Fig. 4 but the primary *y*-axis shows MAE duration for each observer.

Individual and mean MAE durations are plotted as a function of ISI duration in Fig. 5 for both high and low retinal illuminance in open circles and black squares respectively. Note the ordinate scale varies between plots, reflecting individual differences in judged MAE duration. In agreement with the direction discrimination data, the photopic condition first hits its peak duration at an ISIs of between 42 and 83 ms for all subjects and also the group mean. For the scotopic condition the group mean peak motion after-effect occurs at an ISI of 167 ms, slightly longer than the peak ISI for direction discrimination.

### 3.2 Modelling

The normalised model produces output within the range −1 (leftwards energy) to +1 (rightwards energy). Output was computed for a range of *k* values. The goodness-of-fit between the model output and psychophysical data was assessed by calculating the root mean squared (RMS) error for each value of *k*. To perform this calculation the model output and psychophysical data were re-scaled so that they varied over the same range of values. In the case of direction discrimination data, both the data and the model output were re-scaled to the range −0.5 to +0.5, such that 50% performance in the original data (no consistent apparent motion direction, equivalent to zero model output) became zero in the transformed data. For the MAE goodness-of-fit test, each individual’s MAE duration was normalised to the range 0–1 by their own maximum duration in that condition, and similarly the model output was normalised to 0–1 by its maximum output for the particular *k* value. Tables 1 and 2 present the best-fitting *k* values for individuals and the mean for both experiments. The model outputs for the best-fitting *k* values are plotted as dashed lines using the secondary axes in Figs. 4 and 5.

A two-tailed, paired-samples *t*-test was conducted for each experiment comparing the luminance conditions. The best-fitting *k* values for the high luminance condition were significantly higher than those found for the low luminance condition for both the direction discrimination experiment; *t*(4) = 15.263, *p* < .000, and also the MAE experiment; *t*(4) = 9.347, *p* < .001.

## 4 Discussion

Data from two experiments using two different psychophysical measures, under two luminance conditions, reveal that the optimum ISI for perceiving two-stroke apparent motion shifts to longer ISIs under scotopic conditions. The shift in optimum ISI can be modelled as a change in the centre frequency of the biphasic temporal filter serving motion energy sensors.

### 4.1 Comparison of the direction discrimination and MAE duration measurements

The comparison of the psychophysical results using the two measures of motion (Figs. 4 and 5) shows a notable difference in that the direction discrimination data reach a peak ISI followed by a plateau, whereas MAE duration peaks and then declines slowly at longer ISIs. Without the modelling results, one might be tempted to conclude that the high level of performance in direction discrimination at longer ISIs is due to attentive tracking. However, this characteristic is apparent in the model output as well, so it reflects a property of the sensor output. The only difference between the direction discrimination and MAE, reflected in the modelling, was in the stimulus employed. In Direction discrimination we used a single seven-frame cycle of the stimulus, while for MAE we used an extended, continuously cycling sequence (see Fig. 3 for model stimuli). The former may reflect the phasic or transient response of motion sensors, while the latter may reflect tonic or sustained response. Despite the good model fit there is some evidence in Fig. 4 for a ceiling effect in the direction discrimination data; the psychophysical data flattens out at or near 100%, and the model output fluctuates somewhat just below maximum.

### 4.2 High and low luminance data

We previously reported that direction reports for two-stroke motion are optimal at an ISI of 40 ms, but fall to chance using this ISI at low retinal illuminance (Mather & Challinor, 2009). The new data reported here for longer ISI durations reveal that two-stroke motion is not abolished under low luminance conditions, but rather the appearance of unidirectional motion is first experienced at ISI durations at least 80 ms longer than those required at high luminance for both direction discrimination and MAE duration measurements. These findings are in surprisingly close agreement with the 70 ms shift in peak direction discrimination performance found by Takeuchi and De Valois (2009) when luminance decreased for ISI reversals in the retinal periphery. Indeed our results support the evidence for a temporal impulse response function which does not become monophasic at low retinal illuminance, but rather remains biphasic and extends over a longer time period.

### 4.3 Model fitting and central frequencies of the temporal filter

The *k* parameter of the temporal impulse response (Eq. (3)) controls the centre frequency of the temporal filter in the model. Centre frequency was estimated in Matlab by taking the Fourier transform of the impulse response as defined in Eq. (3), and finding the peak of the resulting amplitude spectrum. The group average best-fitting value for *k* at high luminance yields slow and fast filter centre frequencies of 2.5 Hz and 3.0 Hz for direction discrimination and 2.0 Hz and 2.5 Hz for the MAE. These centre frequencies are in close agreement with Pantle’s (1978) measurements of the temporal frequency response characteristics for both MAE cancellations and flicker sensitivity, where he found a peak sensitivity at 2.5 Hz. Our peak values are lower than the values around 8 Hz reported in some other studies (e.g. Snowden, Hess, & Waugh, 1995). Recent work has shown that motion processing involves two temporal band pass channels, a high channel that peaks around 8–12 Hz, and a lower frequency channel with a peak at around 2 Hz (Alais, Verstraten, & Burr, 2005; Cass & Alais, 2006). Our stimuli apparently reflect the operation of the lower frequency channel. We are currently running experiments to see if changes to stimulus parameters allow us to tap the higher frequency channel.

Modelling the data collected at low luminance indicates that a biphasic temporal response operates under scotopic conditions, consistent with the results of others (Sheliga, Chen, FitzGibbon, & Miles, 2006; Snowden et al., 1995; Takeuchi & De Valois, 2009). At low luminance, the group average best-fitting value for *k* yields filter centre frequencies of between 1.0 Hz and 1.5 Hz. This decrease in filter centre frequency as retinal illuminance decreases is consistent with the well-known effect of retinal illuminance on flicker sensitivity (Roufs, 1972). A biphasic temporal filter response allows the visual system to encode rapid motion and to minimise neural motion blur. As retinal illuminance falls there is also a need to capture as much light as possible, so the filter response stretches over a longer time period and therefore peaks at a lower temporal frequency. The same temporal filters that mediate flicker sensitivity appear to provide the input to motion sensors.

## 5 Conclusions

Two different measures of two-stroke apparent motion reveal a similar dependence on ISI duration at different display luminances, consistent with data on the ISI-reversal effect. There is no evidence that attentive tracking contributes to the psychophysical data. The motion-energy model offers a very good account of both the direction discrimination and MAE data. The centre frequency of the best-fitting temporal filters in the model falls at low retinal illuminance but the filter profile remains biphasic, consistent with the known properties of motion and flicker sensitivity.

## Figures and Tables

**Table 1:** Direction discrimination best-fitting *k* values with respective RMS error. Note that ‘Group’ row refers to the model fit for the average of the individual data, whereas the ‘Av Ss’ row gives the average and standard deviation of the model parameters found for individual subjects (i.e. the average of the first five rows in the table).

Direction discrimination				
	High luminance		Low luminance	
	*k*	RMS	*k*	RMS
KLC	110	0.04	60	0.06
BBB	110	0.07	40	0.16
CM	110	0.05	45	0.07
BIH	110	0.06	40	0.15
CNJ	105	0.06	50	0.14
Group	110	0.04	45	0.05
Av Ss	109	0.05	47	0.12
SD	2.24	0.01	8.37	0.05

**Table 2:** Similar to Table 1, but for the MAE experiment.

Motion after-effect				
	High luminance		Low luminance	
	*k*	RMS	*k*	RMS
KLC	85	0.29	55	0.08
BBB	85	0.37	55	0.22
CM	85	0.23	65	0.09
RMH	90	0.05	50	0.15
AIG	90	0.21	55	0.2
Group	90	0.15	55	0.08
Av Ss	87	0.23	56	0.15
SD	2.74	0.12	5.48	0.06
